# Family psittacosis cluster diagnosed by metagenomic next-generation sequencing in Hangzhou City, Eastern China: A case series

**DOI:** 10.1097/MD.0000000000047139

**Published:** 2026-01-09

**Authors:** Zhou Sun, Ke Xu, Liangliang Huo, Xingliang Zhang, Bingbing Chen

**Affiliations:** a Hangzhou Center for Disease Control and Prevention (Hangzhou Health Supervision Institution), Hangzhou, China; b The Affiliated Hospital of Hangzhou Normal University, Hangzhou, China.

**Keywords:** *Chlamydia psittaci*, diagnosis, family cluster, mNGS, psittacosis

## Abstract

**Rationale:**

Psittacosis, a human infection caused by *Chlamydia psittaci* (C psittaci), is often underdiagnosed due to its nonspecific presentation and the limitations of conventional diagnostic methods. This diagnostic challenge can lead to missed outbreaks and delays in appropriate treatment. This report aims to highlight the critical role of metagenomic next-generation sequencing (mNGS) in rapidly identifying *C psittaci* and facilitating the investigation of a family cluster, thereby providing a clearer rationale for its application in similar scenarios.

**Patient concerns:**

In this study, we report a family cluster of psittacosis cases. All affected individuals had a history of direct or indirect contact with backyard poultry during a visit to a rural village before symptom onset. The index case (Case 1) presented with fever and chills and was subsequently hospitalized. The 2 secondary cases (Cases 2 and 3) exhibited similar clinical manifestations and were treated at the same hospital, where doctors promptly collected specimens for testing based on their shared medical history.

**Diagnoses:**

The diagnosis of *C psittaci* pneumonia was confirmed by mNGS analysis of bronchoalveolar lavage fluid obtained from 3 patients through bronchoscopy.

**Interventions:**

Combination therapy involving intravenous moxifloxacin and doxycycline was administered for the treatment of infectious pneumonia.

**Outcomes:**

Following timely diagnosis and targeted antimicrobial therapy, all 3 patients attained full clinical recovery.

**Lessons:**

*C psittaci* pneumonia presents with nonspecific clinical and radiographic features that are indistinguishable from other causes of community-acquired pneumonia. mNGS markedly enhances diagnostic accuracy and shortens the time to diagnosis, proving to be an invaluable tool for early identification and management of outbreaks, particularly in patients with avian or poultry exposure.

## 1. Introduction

*Chlamydia psittaci* (*C psittaci*) is a zoonotic pathogen that affects humans, birds, and various animal populations.^[1]^ The pathogen can be transmitted to humans when the agent is inhaled from bird secretions, dry droplets, or dust on feathers. A variety of clinical manifestations have been documented in humans with psittacosis, ranging from more common subclinical or brief, self-resolving flu-like symptoms to rarer but severe cases of fulminant psittacosis, characterized by multi-organ failure.^[2]^ If pneumonia is present, the radiological findings are often more severe than would be expected based on the clinical signs.

Most human psittacosis cases are sporadic, but outbreaks of psittacosis have also been documented in certain locations.^[3]^ In China, a small number of outbreaks have been reported in recent years, with the number of infected individuals in each outbreak typically limited to 2 to 4.^[4,5]^ However, direct human-to-human transmission is rare. With the advent of metagenomic next-generation sequencing (mNGS) technology in recent years, there has been a notable increase in the identification and reporting of human psittacosis cases.^[6,7]^ In this case report, we describe a comprehensive analysis of the clinical manifestations and laboratory testing of 3 psittacosis cases in a family cluster. The occurrence may be associated with backyard poultry when individuals return to rural areas during the Spring Festival vacation. The diagnosis of psittacosis was confirmed via mNGS, demonstrating the utility of this technique in identifying the etiology of severe pneumonia.

## 2. Case descriptions

### 2.1. Case 1

**Figure 1.**
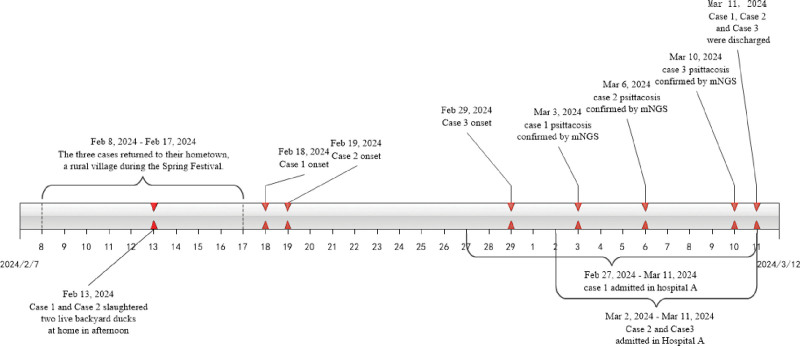
Timeline of exposures, dates of illness onset, hospital admission dates, laboratory findings, and discharge dates. mNGS = metagenomic next-generation sequencing.

The first patient is a 55-year-old unemployed woman who primarily engages in household chores (Table 1). A summary of the patient’s clinical events is presented in Figure 1. On February 27, 2024, she was admitted to the Respiratory Department of the Second People’s Hospital of Yuhang District, Hangzhou City, with a 2-day history of fever accompanied by cough, sputum production, dizziness, and headache. The patient had no prior history of similar symptoms. On admission, the patient was alert and fully oriented (to person, place, and time). Her blood pressure was measured at 100/54 mm Hg, her body temperature was 38.2°C, her heart rate was 113 beats per minute, and her respiratory rate was 19 breaths per minute. Auscultation revealed coarse breath sounds, with no dry or wet rales detected in either lung. No additional significant physical findings were noted during the examination. Serological tests for *Mycoplasma pneumoniae*, *Chlamydia pneumoniae*, adenovirus, influenza virus, parainfluenza virus, and respiratory syncytial virus yielded negative results. Additionally, tests for hepatitis B surface antigen, syphilis antibodies, human immunodeficiency virus antibodies, and hepatitis C antibodies were also negative. Three sets of blood and sputum cultures showed no growth of pathogenic microorganisms, and the patient’s sputum test for the *Mycobacterium tuberculosis* gene was negative. The Widal test for typhoid fever returned negative results for both H and O antibodies. A chest computed tomography (CT) scan revealed multiple patchy opacities in both lungs, indicative of bilateral pneumonia. She was diagnosed with severe community-acquired pneumonia (CAP). Consequently, due to the patient’s symptoms of fever and signs of pulmonary infection, she was admitted and treated with intravenous cefoperazone-sulbactam sodium in combination with levofloxacin for severe CAP. On March 1st, a bronchoscopy was performed, and bronchoalveolar lavage fluid (BALF) was collected for mNGS analysis. After 48 hours, mNGS analysis of BALF identified *C psittaci* (5000 copies/mL) and Rhinovirus A (20,000 copies/mL), confirming the diagnosis of both psittacosis and Rhinovirus A co-infection (Table 2). In accordance with research recommendations, a combination therapy of intravenous moxifloxacin (0.4 g/d) and doxycycline (0.2 g/d) was selected to treat the severe CAP. Following 10 days of targeted anti-infective therapy, the patient achieved afebrile status after the resolution of recurrent fever. Serial chest CT imaging demonstrated progressive improvement, with the day 10 scans revealing a significant reduction in bilateral pulmonary opacities compared to the admission scans. The patient was discharged on the 12th day of hospitalization. A follow-up chest CT obtained 6 weeks post-discharge demonstrated complete resolution of all initially documented pulmonary lesions.

**Table 1:** Clinical characteristics and laboratory data of the 3 cases.

	Case 1	Case 2	Case 3
General data			
Age (yr)	55	52	29
Sex	Female	Male	Female
Data of onset	February 18, 2024	February 19, 2024	February 29, 2024
Outcome	Discharged	Discharged	Discharged
Symptoms			
Maximal temperature (°C)	40.1	38.4	38.5
Headache	Yes	No	No
Cough	Yes	Yes	Yes
Muscle pain	Yes	No	No
Runny nose	No	Yes	Yes
Chest tightness	Yes	Yes	Yes
Pneumonia	Yes	Yes	Yes
Laboratory finding			
WBC (normal: 3.50–9.50 × 10^9^/L)	5.66	12.86	6.33
Neutrophil percentage (normal: 40%–75%)	71.6	88.0	55.8
Lymphocytes percentage (normal: 20%–50%)	21.3	6.3	36.8
hsCRP (normal: <10.00mg/L)	52.88	35.93	41.44
PCT (normal:<0.500ug/L)	0.124	0.058	0.042
PLT (normal: 125–350 × 10^9^/L)	252	183	170
PaO_2_ (normal: 80–110 mm Hg)	57.5	59.8	58.6
PaCO_2_ (normal: 35.0–45.0 mm Hg)	34.1	36.7	37.6

CRP = C-reactive protein, PaCO_2_ = partial pressure of arterial carbon dioxide, PaO_2_ = partial pressure of arterial oxygen, PCT = procalcitonin, PLT = platelet count, WBC = white blood cell.

**Table 2:** Metagenomic sequencing analysis results of BALF obtained from 3 cases.

	Time from onset to test	Type of specimen	Pathogen	Concentration within the specimen (copies/mL)
Case 1	12	BALF	Rhinovirus_A	20,000
			Chlamydia_psittaci	5000
Case 2	5	BALF	Chlamydia_psittaci	10,000
Case 3	8	BALF	Chlamydia_psittaci	3000

BALF = bronchoalveolar lavage fluid.

### 2.2. Case 2

The second patient is the husband of Case 1. The patient presented with acute-onset fever (peak 39°C) on February 19, 2024, accompanied by mild respiratory symptoms, including cough with sputum production (Table 1). After self-administering oral cephalosporin medication, he did not experience significant improvement in his symptoms. Due to sustained fever lasting 10 days and the emergence of new respiratory symptoms, including productive cough with white sputum, chest tightness, and dyspnea, the patient was admitted to the Second People’s Hospital of Yuhang District on March 2 for comprehensive evaluation and treatment. While the patient had a documented history of essential hypertension, there were no preceding instances of comparable clinical manifestations. At presentation, the patient was conscious with normal vital parameters (blood pressure 111/67 mm Hg, heart rate 83 bpm, respiratory rate 20/min, and afebrile at 37°C). Thoracic CT revealed inflammatory changes with lobar distribution affecting both upper lobes and the right lower lobe. He was diagnosed with CAP. In light of his wife’s parallel presentation with similar symptoms, a bronchoscopy was performed on March 4, and BALF was collected for mNGS analysis. After 48 hours, the mNGS analysis detected *C psittaci* sequences at a concentration of 10,000 copies/mL in the BALF, thereby confirming the diagnosis of psittacosis (Table 2). The patient was successfully discharged after 12 days of targeted therapy (Fig. 1).

### 2.3. Case 3

Case 3 involves a 29-year-old woman, the daughter of the patients described in Cases 1 and 2. On February 29, 2024, she developed symptoms including fever, cough, chest tightness, and a runny nose (Table 1). Initially, her clinical presentation was mild, resembling a flu-like illness. However, chest imaging demonstrated right lower lobe consolidation, leading to admission to the Second People’s Hospital of Yuhang District on March 3, 2024. The diagnosis of psittacosis was subsequently confirmed by mNGS on March 10, 2024 (Table 2). Following appropriate treatment, she was discharged after 9 days of hospitalization (Fig. 1).

### 2.4. Exposure history

**Figure 2.**
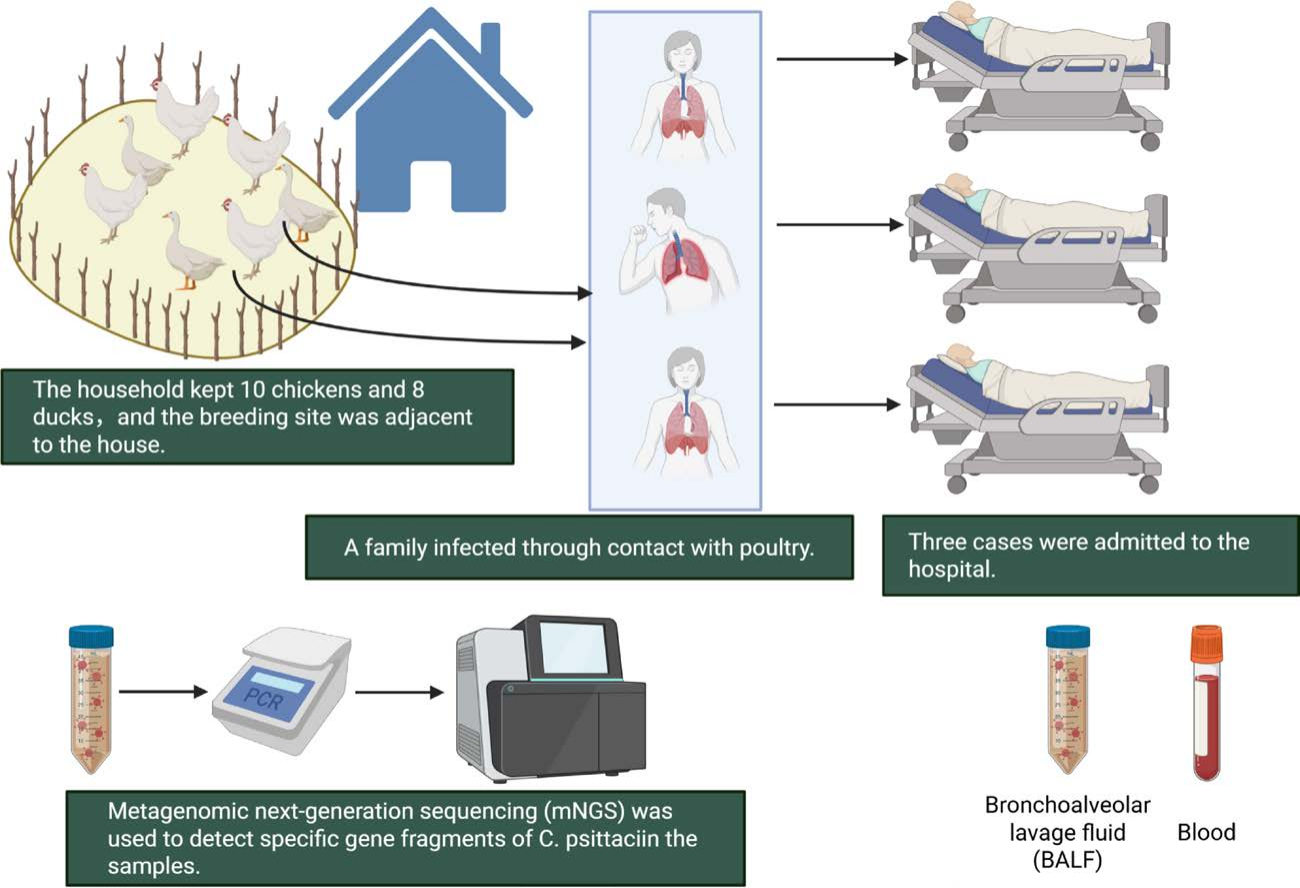
A diagram tracing the transmission of *Chlamydia psittaci* from backyard poultry in a rural village (Yuhang District, Hangzhou City) to 3 family members. BALF = bronchoalveolar lavage fluid, mNGS = metagenomic next-generation sequencing.

The index case (Case 1) resided with her husband (Case 2) and daughter (Case 3) in an urban apartment located more than 200 meters from any parks. None of the 3 patients had a history of smoking or alcohol consumption. Before the Spring Festival, the family returned to their rural hometown in Yuhang District, where their household kept 10 chickens and 8 ducks, cared for by the mother. On February 13, Case 1 and Case 2 prepared 2 ducks for dinner – Case 1 slaughtered the ducks, while Case 2 handled the cleaning. Notably, neither individual used personal protective equipment, such as masks or gloves, while performing these tasks. Case 3 had no direct contact with the poultry before symptom onset. However, her bedroom was in close proximity to the breeding area. During the same period, no other villagers reported febrile or respiratory illnesses. We collected 10 environmental samples from poultry and the poultry environment on March 9, 2019. All samples tested negative for *C psittaci* by PCR. The transmission of *C psittaci* from backyard poultry in a rural village located in Yuhang District, Hangzhou City, to 3 family members is illustrated in Figure 2.

## 3. Results

Three family members were diagnosed with psittacosis via mNGS. Epidemiological investigation revealed exposure to backyard poultry in their rural residence before symptom onset. The index case, a 55-year-old woman with severe CAP, was confirmed by mNGS to have *C psittaci* (5000 copies/mL) and Rhinovirus A co-infection. Her husband and daughter subsequently developed similar symptoms and were confirmed to have psittacosis through mNGS testing. All 3 patients received targeted anti-infective therapy based on the mNGS results. Clinical recovery was successful in all individuals, as evidenced by symptom resolution and improved chest imaging, leading to their discharge after 9 to 12 days of hospitalization.

## 4. Discussion

In this study, we reported a familial outbreak of psittacosis cases confirmed in Hangzhou City, Zhejiang Province, China. Before symptom onset, all 3 affected family members had visited a rural village during the Spring Festival, where they had direct or indirect contact with backyard poultry. The index case (Case 1) presented with fever and chills and was subsequently hospitalized. Chest CT imaging revealed bilateral pulmonary infiltrates, consistent with pneumonia. mNGS of respiratory specimens detected co-infection with *C psittaci* and Rhinovirus. The short incubation period of Rhinovirus (12–72 hours) supports the possibility of nosocomial infection after admission. Exposure from healthcare workers or visitors could have led to co-infection, potentially worsening inflammatory pathology and disease severity. The 2 secondary cases (Cases 2 and 3) exhibited similar clinical manifestations, and mNGS testing confirmed monoinfection with *C psittaci* in both individuals. The laboratory findings in 3 cases, characterized by normal white blood cell counts, alongside markedly elevated C-reactive protein, but normal procalcitonin levels, are noteworthy. This triad of findings helps differentiate it from typical community-acquired bacterial pneumonia, which often presents with pronounced leukocytosis and elevated procalcitonin. Following timely diagnosis and targeted antimicrobial therapy, all 3 patients attained full clinical recovery.

In recent years, the number of reported cases of human psittacosis has increased significantly worldwide.^[8]^ Prior reports of psittacosis outbreaks have been associated with exposure to infected birds such as lovebirds, parrots, and cockatoos, with confirmed cases documented among buyers, hatchery workers, and sales center staff.^[9,10]^ In China, several outbreaks of severe CAP caused by *C psittaci* have also been documented in urban areas, associated with exposure to poultry.^[11]^ This suggests that the main factor for psittacosis in China is contact with poultry, which differs from that in other countries. However, the role of domestic poultry in transmitting *C psittaci* to humans in rural areas remains largely unclear.^[12]^

The spectrum of clinical manifestations associated with psittacosis in humans is broad and highly variable, ranging from asymptomatic infections or mild flu-like symptoms to severe atypical pneumonia, which can, in rare cases, result in fatal outcomes.^[13]^ The clinical presentation of psittacosis often resembles symptoms caused by other infectious agents, such as SARS-CoV-2, seasonal influenza, and other similar pathogens.^[14]^ The main symptoms include fever, cough, dyspnea, and chest tightness, with most cases showing unilateral or bilateral pneumonia.^[15]^ In our study, all 3 cases presented with respiratory symptoms such as fever and cough. They developed unilateral or bilateral pneumonia. Case 1 presented symptoms on February 18th and was diagnosed using the mNGS method on March 1st, resulting in an interval of nearly 2 weeks. Cases 2 and 3 were treated at the same hospital, where doctors promptly collected specimens for testing based on the shared medical history, allowing for the rapid identification of the infectious agent. It was recommended that clinicians obtain a comprehensive medical history and promptly collect samples for testing when evaluating patients with CAP who report recent exposure to avian species or poultry.^[16]^ To mitigate future risks, several measures should be implemented. First, we need to encourage clinicians to test suspected psittacosis cases by using RT-PCR, mNGS, and specific antibodies. Second, we should integrate avian species or poultry exposure as a mandatory Electronic Health Record field for patients with fever or respiratory symptoms to prompt *C psittaci* testing and avoid overlooking potential risk factors. Third, we should create a dedicated “Severe CAP of Unknown Etiology” protocol combining mNGS with atypical pathogen tests (including *C psittaci*) for immediate, parallel testing upon hospital admission, replacing sequential testing to accelerate diagnosis.

## 5. Limitations

This study has several limitations inherent to its focus on a case series. Firstly, the findings were derived from only 3 related cases within a single family cluster. While their distinctive exposure history of slaughtering poultry strongly suggests the source of infection, the small and unique nature of this cluster means we cannot definitively rule out other common, undetected exposure sources, and the findings may not represent typical psittacosis transmission. Secondly, the diagnosis in all cases was reliant solely on mNGS, a highly sensitive but costly and nonroutine technique. The absence of parallel confirmatory testing using traditional methods, such as PCR or serology, prevents us from evaluating the comparative performance of these diagnostics in this context.

## 6. Conclusion

In conclusion, we reported a family cluster infection caused by *C psittaci* utilizing mNGS for etiological diagnosis. We described the complete and precise procedures of the infection route, clinical manifestations, diagnosis, and treatment. A notable limitation was the delayed application of mNGS testing, which was not performed until 14 days after symptom onset, despite the patients’ clear history of poultry exposure. This delay highlights the need for improved clinical awareness and timely utilization of advanced diagnostic tools in cases of suspected zoonotic pneumonia. Early detection and intervention are critical for preventing human transmission and improving clinical outcomes.

## Author contributions

**Data curation:** Ke Xu, Liangliang Huo, Xingliang Zhang.

**Writing – original draft:** Zhou Sun.

**Writing – review & editing:** Bingbing Chen.
